# The significance of epithelial–mesenchymal transition (EMT) in the initiation, plasticity, and treatment of glioblastoma

**DOI:** 10.1016/j.gendis.2025.101711

**Published:** 2025-06-06

**Authors:** Pu Xia

**Affiliations:** a Biological Anthropology Institute, College of Basic Medical Science, Jinzhou Medical University, Jinzhou, Liaoning 121001, China; b OncoRay-National Center for Radiation Research in Oncology, Faculty of Medicine and University Hospital Carl Gustav Carus, Technische Universität Dresden and Helmholtz-Zentrum Dresden-Rossendorf, Dresden 01309, Germany

**Keywords:** Cancer stem cell, Epithelial‒mesenchymal transition, Glioblastoma, Plasticity, Treatment

## Abstract

Epithelial‒mesenchymal transition (EMT) is a dynamic cellular process in which epithelial cells lose their characteristics and acquire mesenchymal traits, leading to enhanced migratory, invasive, and stem-like properties. EMT is a fundamental mechanism in cancer progression, including in glioblastoma (GBM), an aggressive brain tumor known for its poor prognosis and resistance to treatment. In GBM, EMT has been implicated in tumor initiation, plasticity, metastasis, and treatment resistance, making it a key factor in the pathophysiology of the disease. The process of EMT can promote tumor cell migration and invasion, facilitating the spread of cancer cells within the brain. Additionally, EMT is believed to contribute to the maintenance of cancer stem cells, which are thought to be responsible for tumor recurrence and resistance to conventional therapies. Given these multifaceted roles, understanding the molecular pathways and regulatory networks that drive EMT in GBM is critical for identifying new therapeutic targets. This review summarized the roles of EMT in GBM initiation and progression, its impact on cancer cell behavior, and the challenges of targeting EMT in therapy, highlighting potential strategies to overcome resistance and improve treatment outcomes.

## Introduction

Glioblastoma (GBM), a malignant tumor originating from glial cells in the neuroectoderm, is the most common primary central nervous system tumor in humans and the most aggressive form of glioma.^1^ Patients typically present with symptoms such as headache, nausea, vomiting, seizures, and visual disturbances.^2^ The exact etiology and pathogenesis of GBM remain incompletely understood, although two major risk factors—radiation exposure and genetic predisposition—have been identified.^3^ Additionally, occupational hazards and lifestyle factors are considered significant contributors to the development of the disease.^3^ The malignant progression and high recurrence rate of GBM are key reasons why it remains difficult to treat effectively.^4^ Surgical resection is the primary treatment for gliomas, and postoperative radiotherapy is often used as an adjunctive measure with proven efficacy.^5^ Advances in imaging technology, surgical techniques, radiotherapy, and the development of new chemotherapy agents have significantly improved the prognosis for glioma patients in recent years.^6^ However, despite these advances, gliomas, particularly malignant variants like GBM, remain highly invasive, and no definitive cure currently exists.^7^ As a result, the prognosis for most patients remains poor, with a 5-year survival rate of approximately 25%, even with comprehensive treatment regimens.^7^

Epithelial‒mesenchymal transition (EMT) is a biological process in which epithelial cells undergo transformation into cells with a mesenchymal phenotype through a tightly regulated program.^8^ EMT plays a crucial role in various physiological and pathological processes, including embryonic development, chronic inflammation, tissue remodeling, cancer metastasis, and fibrotic diseases.^8^ During EMT, epithelial cells lose their polarity and adhesion to the basement membrane, acquiring mesenchymal traits, such as increased migratory and invasive capabilities, resistance to apoptosis, and the ability to degrade the extracellular matrix.^9^ EMT is a critical mechanism by which epithelial-derived cancer cells gain the ability to migrate and invade surrounding tissues.^10^ The activation of EMT is a key step in cancer cell metastasis, during which epithelial cells acquire mesenchymal characteristics, increasing their motility and invasive potential.^10^ Key markers of EMT include the loss of epithelial markers, such as E-cadherin and cytokeratins, alongside the up-regulation of mesenchymal markers like N-cadherin, vimentin, and fibronectin.^11^ These changes in marker expression lead to a reduction in cell–cell adhesion between transitioning epithelial cells and adjacent cells, while simultaneously increasing the secretion of enzymes that degrade the extracellular matrix.^11^ The down-regulation of E-cadherin expression is a central event in EMT and cancer metastasis, and it has become a molecular hallmark of cancerous EMT.^12^ The loss of E-cadherin is often accompanied by increased expression of N-cadherin, a critical factor for tumor cell invasiveness.^12^ Additionally, the overexpression of vimentin has been linked to enhanced invasiveness and metastasis in various cancers, including GBM.^12^ EMT-inducing transcription factors belong to three distinct protein families: the SNAIL family (SNAIL and Slug), the ZEB family (ZEB1 and ZEB2), and the basic helix‒loop‒helix (bHLH) family (TWIST1, TWIST2, and TCF3).^13^ These transcriptional regulators control the expression of EMT-related genes by activating or inhibiting their promoters.^13^

## Significance of EMT in GBM

### Evidence supporting the occurrence of EMT in GBM

The role of EMT in epithelial tumors has been extensively studied in various cancers, including breast, colorectal, pancreatic, thyroid, and lung cancers.^14^, ^15^, ^16^, ^17^, ^18^ EMT is a key process by which progressive epithelial tumors acquire the ability to invade, metastasize, and resist treatment.^19^ However, the occurrence of EMT in malignant gliomas has remained a subject of debate, primarily due to the absence of typical epithelial characteristics in glioma cells.^20^

E-cadherin expression has been found to be similar across high-grade and low-grade gliomas as well as in normal brain tissue, which has led to skepticism regarding the relevance of EMT in gliomas.^21^ Interestingly, the expression of E-cadherin in gliomas may reflect a unique pattern of EMT, distinct from what is typically seen in epithelial cancers.^21^ Dysregulation of E-cadherin expression has been observed in invasive glioma phenotypes, with decreased E-cadherin levels potentially reducing tumor migration and increased expression correlating with enhanced invasiveness—an observation that contrasts with the role of E-cadherin in epithelial tumors.^22^ The transcription factor TWIST1 plays a core role in mediating EMT by inhibiting E-cadherin and promoting N-cadherin expression.^13^ GBM cells overexpressing TWIST1 do not undergo the typical transition from E-cadherin to N-cadherin 23. This suggests that the E-cadherin to N-cadherin switch is not required to drive the invasive stromal phenotype of GBM. Although the expression pattern of E-cadherin during EMT in gliomas differs from that observed in epithelial tumors, there is a clear connection between EMT and the invasion and growth of malignant gliomas.^24^ The interstitial changes observed in GBM are closely tied to its aggressive clinical phenotype, further suggesting that the EMT process may play a crucial role in the invasive behavior of GBM.^25^ Gene overexpression associated with interstitial tissue remodeling has been detected in GBM biopsy specimens, indicating that primary GBM exhibits interstitial properties and invasive characteristics.^26^ The role of N-cadherin in the invasive behavior of malignant gliomas remains a subject of ongoing investigation, with inconsistent findings in the literature. While N-cadherin is generally expressed at low levels in human glioma samples and glioma cell lines, its down-regulation has been shown to promote glioma cell migration and invasion.^27^ Conversely, the up-regulation of N-cadherin may be linked to the restoration of cell polarity and the inhibition of migration, further complicating its role in glioma invasion.^28^

Unlike epithelial cancers, GBM cells do not display distinct epithelial markers, such as E-cadherin, and instead exhibit a more plastic and heterogeneous phenotype that facilitates the transition to a mesenchymal-like state. In GBM, EMT is characterized by the up-regulation of mesenchymal markers, as well as transcription factors, which are often associated with poor prognosis. To better understand the role of the EMT process in GBM, the expression levels of EMT-related genes, such as N-cadherin, E-cadherin, and vimentin, were analyzed, along with their prognostic significance, using the R programs “Limma” and “Survival”. The analysis revealed that both N-cadherin and vimentin were overexpressed in tumor tissues compared to normal tissues, whereas E-cadherin expression did not differ significantly between the two tissue types (Fig. S1A). Consistent with these findings, high expression levels of N-cadherin and vimentin were associated with poor prognosis in GBM patients (Fig. S1B). An EMT-related prognostic signature (EMTscore) was then calculated for studies published between 2005 and 2020. The EMTscore was derived from EMT/MET gene signatures identified in the literature and was computed using gene set enrichment analysis (GSEA) method. The association between the EMTscore and overall survival (OS) and progression-free interval (PFI) was assessed using Kaplan–Meier analysis. As shown in the survival forest plot, a high EMTscore was identified as a significant risk factor for poor OS in GBM patients across all studies with statistically significant results (Fig. S2A). However, in contrast to most studies, two reports observed a negative correlation between the EMTscore and PFI in GBM patients (Fig. S2B). On the basis of the results above, EMT transformation generally appears to be a disadvantageous factor for glioma patients.

### Signaling pathways are involved in GBM cells during EMT

**Figure 1:**
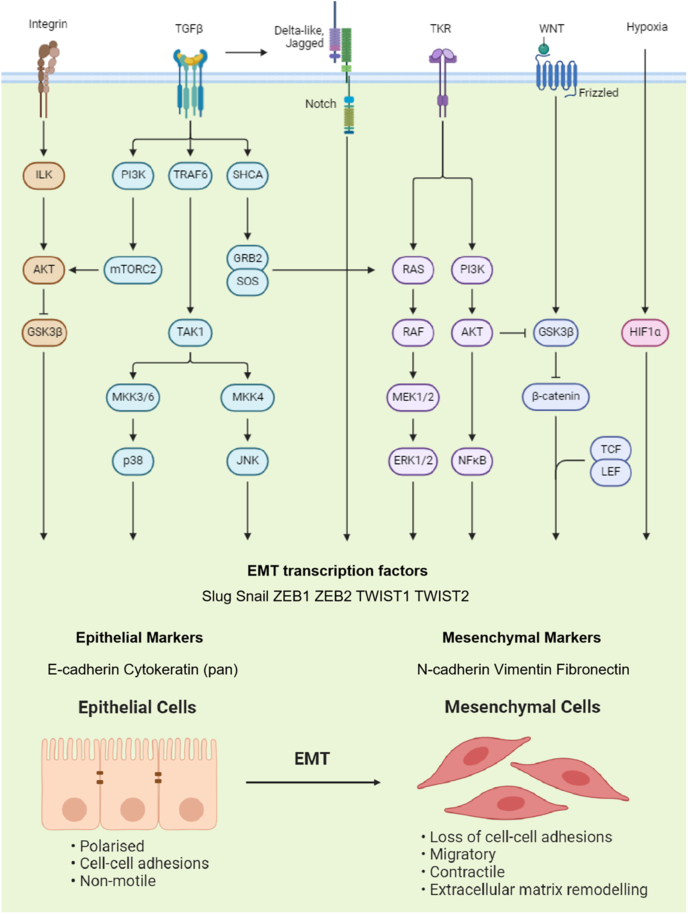
Diagram illustrating the epithelial–mesenchymal transition (EMT)-associated signaling pathways involved in the proliferation, invasion, and stemness of glioblastoma (GBM) cells. Major signaling pathways, including the TGF-β, Wnt/β-catenin, Integrin, and others, are shown to be critical mediators of EMT transcription factors.

The EMT in GBM is regulated primarily by several key signaling pathways, including the TGF-β, PI3K/Akt, Notch, Wnt, and hypoxia-inducible factor pathways^29^, ^30^, ^31^, ^32^, ^33^, ^34^ (Fig. 1).

**Figure 2:**
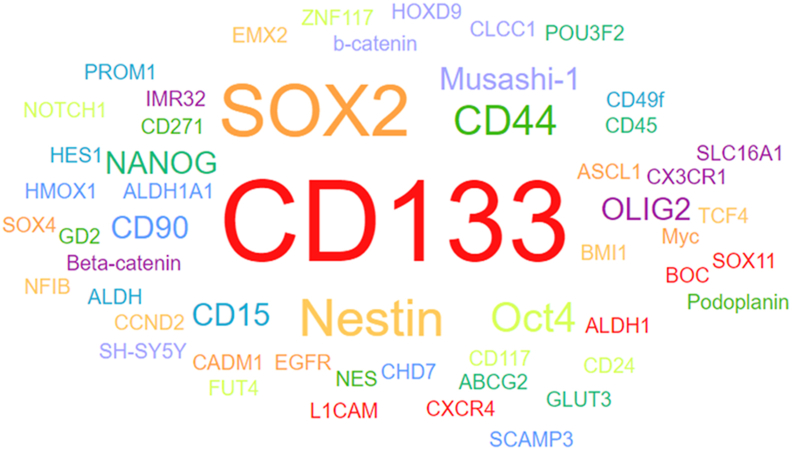
Word cloud visualizing the surface markers of glioblastoma stem cells (GSCs) generated using CellMarker 2.0. This visualization highlights the key cell surface markers that are commonly associated with GSCs, which play a crucial role in the initiation, progression, and recurrence of GBM. The size of each term in the word cloud corresponds to the frequency or significance of the respective marker in GSC populations.

TGF-β plays a crucial role as a multifunctional regulator of cell growth, apoptosis, differentiation, and migration, exerting a dual effects on tumor progression.^35^ In the early stages of tumor development, TGF-β acts to suppress tumorigenesis by inducing growth arrest and promoting apoptosis.^35^ However, in advanced cancer stages, TGF-β facilitates tumor progression, invasion, and migration by triggering EMT.^35^ TGF-β initiates its effects by binding to specific receptors, which leads to the activation of the Smad2 and Smad3 proteins.^36^ These Smads then associate with Smad4 in the cytoplasm to form a trimeric complex, which subsequently translocates into the nucleus.^37^ The complex directly activates the transcription of EMT-related factors, such as SNAIL, Slug, ZEB1, ZEB2, and TWIST, thereby promoting EMT and enhancing cell invasion and metastasis.^37^ In GBM, TGF-β induces EMT via the TGF-β‒Smad2 signaling pathway, as well as through the 3-phosphoinositide-dependent kinase 1 (PDK1)/c-Jun pathway, which contributes to cellular invasion and migration.^38^^,^^39^ Additional factors that modulate TGF-β-induced EMT in GBM have been identified. For instance, the overexpression of the neurotrophic factor prosaposin (PSAP) promotes GBM cell invasion and EMT through the TGF-β1/Smad signaling cascade.^40^ Likewise, fibronectin, a glycoprotein, facilitates malignant progression in gliomas via the TGF-β-induced EMT pathway.^41^ The HOX gene family, including transcription factors HOXC6 and HOXA13, serves as a prognostic marker for disease-free survival (DFS) and OS in GBM patients, while also driving EMT in GBM cells through the TGF-β pathway.^42^^,^^43^ Furthermore, elevated expression of sodium‒potassium‒chloride cotransporter isoform 1 (NKCC1) is associated with poor prognosis in GBM patients and is linked to increased levels of CDH2 and vimentin, both of which are regulated by the TGF-β signaling pathway.^44^ Claudin-3 (CLDN3), which is up-regulated in GBM, also plays a role in promoting tumor cell growth and EMT via TGF-β signaling.^45^ Hematopoietic cell kinase (HCK), a member of the Src family of protein tyrosine kinases (SFKs), amplifies TGF-β-induced EMT in GBM cells.^46^ Additionally, polycomb repressive complex 2 (PRC2), an important epigenetic modifier in GBM, regulates the EZH2/miR-490/TGFB-induced factor homeobox 2 (TGIF2) axis to activate TGF-β signaling, thus promoting cell migration and EMT.^47^ Microtubule associated monooxygenase, calponin and LIM domain containing 2 (MICAL2), which interacts with the TGF receptor type I (TGFRI), enhances both the proliferation and EMT of GBM cells through the TGF-β/p-Smad2 signaling pathway.^48^

Dysregulation of the Wnt/β-catenin signaling pathway is a critical driver of EMT, which is characterized by the nuclear translocation of β-catenin and the down-regulation of E-cadherin 49. As a central component of the Wnt signaling cascade, β-catenin accumulation in the cytoplasm leads to its subsequent translocation to the nucleus, where it activates the transcription of genes related to EMT.^49^ In the nucleus, β-catenin forms a complex with T cell factor/lymphoid enhancer factor (TCF/LEF), which then promotes the transcription of key EMT regulators, such as TWIST and SNAIL, thereby driving the process of EMT.^49^ In the early stages of GBM, elevated expression of the epidermal growth factor receptor (EGFR) leads to β-catenin phosphorylation, which enhances its binding to calcium-binding proteins or increases its expression, subsequently facilitating glioma cell migration and EMT.^50^ Furthermore, activation of the Wnt/β-catenin signaling pathway has been shown to promote the expression of ZEB1 and further induce EMT in GBM, thereby exacerbating its migratory and invasive potential.^51^ Secreted frizzled-related protein 2 (SFRP2) plays a protective role by inhibiting GBM invasion, migration, and EMT. It achieves this by reducing the levels of phosphorylated β-catenin (p-β-catenin) and matrix metalloproteinase 2 (MMP2).^52^ Conversely, Wnt-5a modulates MMP-2 synthesis through β-catenin signaling, enhancing glioma cell migration.^53^ Epsin 3 (EPN3) has been shown to promote GBM cell migration and invasion by activating transcription factors such as Slug, TWIST, and ZEB1, which induce EMT through both the Notch and Wnt/β-catenin signaling pathways.^54^ In contrast, spalt-like transcription factor 1 (SALL1) suppresses cell migration by preventing EMT through the inhibition of Wnt/β-catenin signaling.^55^ SWI/SNF-related matrix-associated actin-dependent regulator of chromatin subfamily C member 2 (SMARCC2) negatively regulates Wnt/β-catenin signaling by controlling c-Myc expression, thereby reducing glioma cell invasion and migration.^56^ Similarly, Golgi membrane protein 1 (GOLM1), a type II transmembrane protein located in the cis- and medial-Golgi, promotes migration, invasion, and EMT through the Wnt/β-catenin pathway.^57^ Down-regulation of tumor protein D52-like 2 (TPD52L2) has been shown to enhance cell invasion by up-regulating β-catenin and SNAIL, both of which are key mediators of EMT.^58^ Additionally, budding uninhibited by benzimidazole 1 (BUB1) triggers EMT in GBM cells by activating the Wnt/β-catenin pathway.^59^ On the other hand, down-regulation of signal transducer and activator of transcription 1 (STAT1) can reduce the aggressiveness of GBM cells by inhibiting the Wnt/β-catenin-mediated EMT pathway.^60^ Forkhead box protein M1 (FOXM1) promotes GBM progression by up-regulating EMT, invasion, angiogenesis, and Wnt/β-catenin signaling.^61^ Moreover, the PRMT6-YTHDF2-Wnt-β-catenin axis has been shown to facilitate GBM migration, invasion, and EMT both *in vitro* and *in vivo*.^62^

The Notch signaling pathway plays a complex role in gliomas, acting as both an oncogene and a tumor suppressor depending on the context.^63^ On the one hand, Notch can promote EMT by transcriptionally regulating key signaling molecules, such as SNAIL and TWIST. This leads to the suppression of E-cadherin expression and the up-regulation of N-cadherin and vimentin, thereby enhancing the migration and invasion capabilities of tumor cells.^64^ Additionally, Notch signaling regulates the interaction between glioma stem cells and the tumor microenvironment, increasing their stemness and invasive properties, which ultimately contributes to tumor progression.^65^ Interestingly, in a glioma mouse model with Notch gene knockout, the absence of Notch signaling accelerated both glioma formation and progression, suggesting that Notch may exert tumor-suppressive effects in GBM.^66^ In glioblastoma stem cells expressing high levels of the proneural transcription factor achaete-scute complex-like 1 (ASCL1), Notch signaling collaborates with the Wnt/β-catenin pathway to inhibit cellular differentiation and promote proliferation, driving malignant tumor progression.^67^ Furthermore, TGF-β signaling can induce high expression of Notch ligands, such as Jagged1, and inhibition of Notch pathway components has been shown to block TGF-β-mediated EMT induction.^68^ Additionally, the overexpression of Notch1 has been shown to activate the AKT pathway, which subsequently promotes the nuclear translocation of β-catenin and NF-κB.^69^ This, along with Notch-mediated overexpression of SNAIL, ZEB1, and vimentin, further enhances glioma cell invasion and migration.^69^

The PI3K/Akt/GSK-3β signaling pathway plays a critical role in regulating glioma cell proliferation, stemness maintenance, and tumor growth.^70^ High levels of phosphorylated Akt lead to the phosphorylation and subsequent inactivation of GSK-3β.^70^ The inactivation of GSK-3β facilitates the accumulation of β-catenin in the nucleus, where it promotes EMT and enhances glioma cell proliferation and invasion.^70^ Inhibition of the PI3K/Akt signaling pathway via specific PI3K and Akt inhibitors has been shown to significantly reduce the invasiveness of glioma cells.^71^ This is accompanied by a down-regulation of vimentin expression and an up-regulation of E-cadherin expression, further suggesting a reversal of EMT processes.^71^ Additionally, knockout of translocation-associated membrane protein 2 (TRAM2) in GBM cells leads to a significant reduction in cell proliferation, migration, and invasion, as well as a marked inhibition of EMT.^72^ Importantly, activation of PI3K can reverse the effects of TRAM2 on EMT in GBM cells, highlighting the functional interplay between PI3K signaling and TRAM2 in regulating EMT.^72^ Moreover, knocking out eukaryotic translation elongation factor 1D (EEF1D) in GBM cells results in increased expression of E-cadherin, whereas the expression of N-cadherin, SNAIL, and β-catenin significantly decreases.^73^ Along with these changes, the expression of PI3K, phosphorylated PI3K, Akt, and phosphorylated Akt is also significantly reduced, further indicating the involvement of the PI3K/Akt pathway in regulating EMT and the progression of GBM.^73^

### The effect of hypoxia on EMT in glioma cells

The hypoxic microenvironment plays a critical role in inducing EMT in GBM cells, as evidenced by morphological changes and enhanced invasion and migration capabilities.^74^ The stabilization and activation of hypoxia-inducible factor 1α (HIF-1α) promote glioma cell proliferation, angiogenesis, and EMT, thereby contributing to tumor metastasis through the activation of GLI family zinc finger 1 (GLI1).^75^^,^^76^ Under hypoxic conditions, knockdown of ZEB1 in glioma cells significantly reduces their invasiveness, with no major changes observed in stromal markers.^77^ This suggests that ZEB1 plays a key role in mediating hypoxia-induced EMT in GBM.^77^ Furthermore, the up-regulation of EMT-related transcription factors, such as ZEB1, ZEB2, SNAIL1, Slug, and TWIST has been observed in GBM patients following treatment with bevacizumab, a humanized monoclonal antibody that targets vascular endothelial growth factor (VEGF).^78^ Interestingly, inhibition of HIF-1α or HIF-2α attenuates the mesenchymal phenotype of GBM cells, suggesting that HIFs are crucial regulators of EMT in response to hypoxia.^78^ In addition to HIF signaling, the Hedgehog pathway also plays a significant role in hypoxia-induced glioma metastasis by regulating EMT through smoothened and GLI1.^79^ Inhibition of the Hedgehog signaling pathway has been shown to suppress glioma cell invasion, which is associated with the reduced expression of VEGF, a key mediator of angiogenesis.^80^

### The roles of EMT in glioblastoma stem cells (GSCs)

Cancer stem cells (CSCs) are characterized by their self-renewal capacity, multi-lineage differentiation potential, and resistance to therapy.^81^, ^82^, ^83^ GSCs, a subtype of CSCs, play a key role in tumor initiation and exhibit significant heterogeneity within gliomas.^83^ In addition to the typical traits of CSCs, glioma stem cells possess the ability to promote tumor angiogenesis, enhance tumor invasion, and exhibit a heightened resistance to both radiotherapy and chemotherapy.^84^ These properties contribute to the rapid recurrence of gliomas after treatment.

### Glioblastoma stem cell markers (Fig. 2)

The CD133^+^ subset of glioma cells is known for its strong self-renewal and proliferation abilities.^85^, ^86^, ^87^ Compared to CD133^-^ cells, CD133^+^ GSCs contribute significantly to tumor metastasis, recurrence, and resistance to chemotherapy, thereby accelerating glioma progression and leading to a poor prognosis [85–87]. Additionally, in response to irradiation, CD133^+^ glioma cells exhibit a stronger DNA repair ability than CD133^-^ cells do.^88^ This enhanced DNA repair is attributed to increased expression levels of key proteins involved in the DNA damage response, including ATM, Rad17, Chk1, and Chk2.^88^

Stage-specific embryonic antigen-1 (SSEA-1, also known as LeX/CD15) is an embryonic antigen found in embryonic tissue, the adult central nervous system, and the GBM.^89^^,^^90^ SSEA-1^+^ cells can be present in CD133^-^ GBM tissues, and SSEA-1+ cells have emerged as novel markers, particularly for identifying CD133^-^ GSCs.^90^ Compared to CD133, SSEA-1 offers broader applicability for GBM research and diagnostics.^90^ SSEA-1^+^ GBM cells exhibit significant tumorigenic potential; when implanted into the skulls of nude mice, these cells form new glioma tissue.^91^ Additionally, SSEA-1^+^ cells demonstrate clonogenic potential, suggesting that SSEA-1 may serve as a target for proliferating tumor cells in the brain 91. Interestingly, high clonogenicity has been observed in both CD133^-^/CD15^-^ and CD133^+^/CD15^+^ GBM cells, but not in CD133^-^/CD15^+^ cells.^92^ This finding highlights the complex heterogeneity of GBM, suggesting that relying on a single marker like CD133 may not be sufficient for the definitive identification of GSCs across all GBM subtypes.

Nestin is an intermediate filament protein and a key marker of neural precursor cells, specifically expressed in proliferating cells during embryonic development.^93^ It plays a crucial role in the formation of the cytoskeleton, signal transduction, organ development, and the regulation of cell metabolism, proliferation, and differentiation.^94^ Nestin is expressed in both low-grade and high-grade gliomas,^95^^,^^96^ and its increased expression is associated with increased glioma malignancy, leading to poorer patient survival rates.^95^^,^^96^ In GSCs, Nestin is coexpressed with other stem cell markers, including CD133, Musashi-1, and Sox-2.^97^ In GSC xenograft models, both proliferating cell nuclear antigen (PCNA) and Nestin expression are positive, with Nestin levels showing a positive correlation with the degree of differentiation, malignancy, migration, and invasion of glioma cells.^98^

A2B5 is a glycosphingolipid molecule expressed on the surface of oligodendrocyte precursor cells.^99^ In glioma, A2B5^+^/CD133^-^ cells exhibit increased self-renewal, tumor formation, and migratory and invasive capabilities compared to A2B5^-^/CD133^-^ cells.^100^ These properties are believed to contribute significantly to glioma recurrence.^100^ Furthermore, the presence of the A2B5 molecule is associated with poor prognosis, low differentiation, and a high recurrence rate in glioma patients.^101^

BMI1, a member of the polycomb group family, plays a critical role in embryonic development, particularly in skeletal and structural formation, as well as in the development of the nervous system.^102^^,^^103^ It is also involved in transmitting signals related to self-renewal and DNA damage in both normal and tumor cells.^104^ Downregulation of BMI1 has been shown to inhibit glioma cell proliferation, decrease telomerase expression, and increase sensitivity to chemotherapy.^105^^,^^106^ Additionally, BMI1 enhances the migration and invasion of glioma cells through pathways, such as the NF-κB and MMP-9 pathways.^107^ BMI1 expression levels are significantly higher in glioma tissue than in normal brain tissue; however, BMI1 alone cannot be used as a reliable indicator of patient survival.^108^ BMI1 is highly expressed in CD133^+^ GBM cells, where it is essential for their self-renewal.^109^^,^^110^ This effect occurs independently of the INK4A(p16)/ARF(p14)/p53 pathway and helps prevent apoptosis and/or differentiation into neurons and astrocytes.^109^^,^^110^

LGR5, also known as G protein-coupled receptor 49 (GPR49) or GPR67, is a member of the GPCR protein family.^111^ It is widely expressed in various tissues, including muscle, placenta, breast, spinal cord, and brain, and serves as an important marker for adult stem cells.^112^ LGR5 expression is up-regulated through the Wnt signaling pathway, which plays a crucial role in brain development by regulating the expansion and maintenance of neural stem and precursor cell populations.^113^ In GBM, LGR5 is considered a marker of poor prognosis and is essential for the survival of GSCs.^114^ It promotes glioma invasion, migration, and EMT by activating the Wnt/β-catenin signaling pathway.^115^ Notably, high expression of LGR5 is associated with elevated levels of oligodendrocyte transcription factor 2 (OLIG2), a proneural factor that is crucial for both neurogenesis and the maintenance of GSCs.^116^

### EMT-induced stemness in GBM cells

EMT is not only linked to tumor invasion, metastasis, recurrence, and treatment resistance but also plays a critical role in enabling tumor cells to acquire stem cell-like characteristics.

Knocking out ZEB1 in GBM cells inhibits the expression of key glioma stem cell markers, including CD133, SOX2, and OLIG2, thereby suppressing GSC initiation, invasion, and chemoresistance.^117^ Similarly, inhibiting the expression of TWIST significantly reduces both the growth and sphere formation capabilities of GSCs.^23^^,^^118^ On the other hand, the overexpression of the oncogenic factor KAI1 C-terminal interacting tetraspanin (KITENIN) promotes EMT in glioma cells by increasing the expression of EMT markers, such as N-cadherin, ZEB1, ZEB2, SNAIL, and Slug. KITENIN also up-regulates the expression of GSC markers, such as CD133, ALDH1, and erythropoietin-producing hepatocellular receptor B1 (EPH-B1), thereby enhancing the ability of these cells to form neurospheres and clones.^119^ Similarly, the overexpression of V-set and immunoglobulin domain containing 4 (VSIG4) induces EMT in glioma cells, significantly promoting invasion and migration.^120^ This is accompanied by an increase in the number of GSC neurospheres and the up-regulation of stemness markers, such as CD133, EZH2, c-Met, and CD44.^120^ Notably, the microRNA Let-7g-5p suppresses EMT by down-regulating VSIG4 expression, while also reducing GSC formation.^120^ In addition, a reduction in CD73 protein levels leads to decreased SNAIL1 expression, thereby significantly inhibiting GSC survival, proliferation, and colony-forming ability, while impairing downstream adenosine (ADO) signaling.^121^ S100 calcium-binding protein A4 (S100A4), also known as FSP1 or metastasin, is a small calcium-binding protein that regulates the expression of SNAIL2 and ZEB in glioma cells.^122^ Glioma cells that express S100A4 demonstrate tumor-initiating and sphere-forming capabilities.^122^ Treatment with β-elemene in glioma cells results in down-regulation of N-cadherin and β-catenin, while up-regulating E-cadherin and decreasing CD133 expression, which suggests a reduction in GSC-like properties.^123^ Additionally, the diterpene carnosol, an inhibitor of the MDM2/p53 complex, decreases sphere formation and promotes GSC apoptosis by inhibiting EMT through reduced expression of Slug, SNAIL, TWIST, and ZEB1.^124^ The diterpene carnosol also interferes with GSC self-renewal by down-regulating key stemness-related genes, such as Nanog, SOX2, and Oct4.^125^ Moreover, co-culture of human umbilical cord blood stem cells (hUCBSCs) with CD133^+^ glioma cells results in the down-regulation of several EMT-related markers, including N-cadherin, β-catenin, vimentin, TWIST1, and SOX2, while up-regulating E-cadherin 125. Interestingly, glioma cells cultured in 3D bioprinted scaffolds exhibit increased expression of CD133 and Nestin 126. Furthermore, the levels of SNAIL and TWIST increased in a time-dependent manner, further supporting the idea that glioma cells undergoing EMT acquire stem cell-like characteristics.^126^

**Figure 3:**
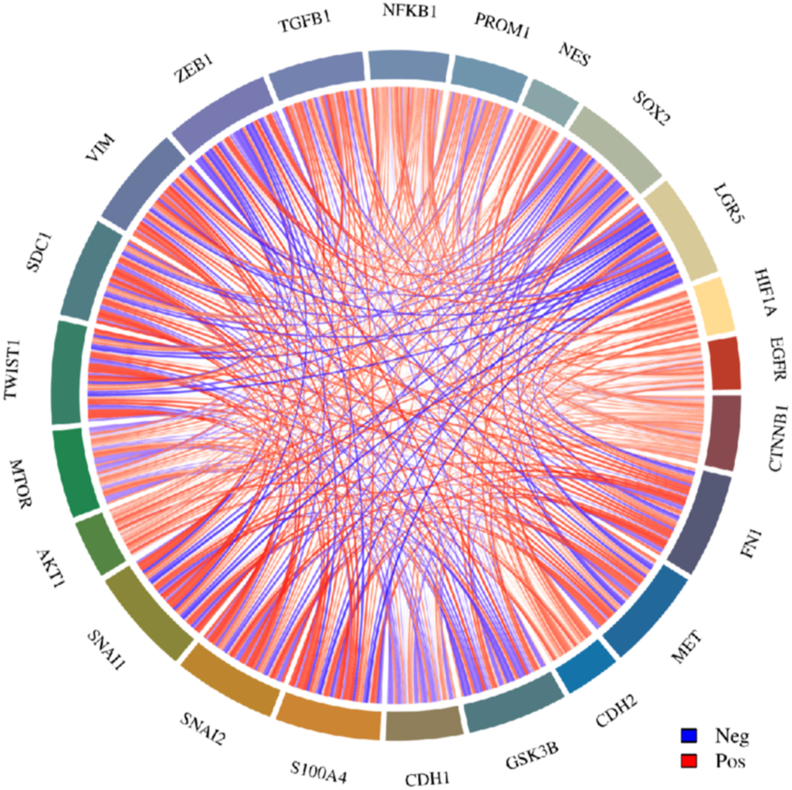
Multi-gene correlation map of EMT-related genes and GSC markers generated using the R software package “ggstatsplot” and visualized with the “pheatmap” package. This heatmap presents the correlation patterns between key EMT-associated genes and surface markers of GSCs, offering insights into the molecular interactions that underpin glioblastoma stem cell biology. The color intensity in the heatmap represents the strength of the correlation, with positive and negative correlations clearly indicated.

Taken together, these findings underscore the complex interplay between EMT, GSC properties, and the regulation of stemness markers in glioma cells. These findings highlight potential therapeutic strategies targeting EMT-related pathways to inhibit GSC formation and progression in GBM. A multi-gene correlation map of EMT-related genes and stemness markers in GBM is shown in Figure 3, with detailed information provided in Figure S3.

### Potential approaches for developing EMT-targeted therapies

Targeted inhibition of EMT offers a promising strategy to prevent tumor cell invasion and metastasis, eliminate CSCs, and overcome drug resistance. Currently, several approaches are being explored to specifically target EMT, including inhibiting EMT-inducing transcription factors, targeting key EMT signaling pathways, modulating non-coding RNAs associated with EMT, and identifying EMT-related biomarkers.

### Natural products

Calycosin has been shown to inhibit the migration and invasion of GBM cells by suppressing TGF-β-mediated EMT.^127^ Astragaloside IV (AS-IV) blocks Wnt/β-catenin signaling, which is involved in EMT in glioma cells, thereby reducing their invasive and migratory capabilities.^128^ Melatonin suppresses glioma metastasis under hypoxic conditions by inhibiting TGF-β/Smad-C-C motif chemokine ligand 20 (CCL20) activity and modulating EMT through the activation of Smad7.^129^ Luteolin reverses EMT by inducing cytoskeletal changes, up-regulating the epithelial marker E-cadherin, and down-regulating mesenchymal markers such as N-cadherin, SNAIL, and vimentin 130. Additionally, luteolin increases intracellular reactive oxygen species levels, triggering endoplasmic reticulum stress and mitochondrial dysfunction, which contribute to its anti-EMT effects in GBM cells.^131^ Verbascoside (VB) inhibits the expression of TGF-β, vimentin, and SNAIL and down-regulates c-Met and ZEB1, thus suppressing GBM proliferation, migration, and invasion.^132^ Resveratrol inhibits EMT in GBM cells through regulation of both the Smad and Wnt signaling pathways.^133^^,^^134^ Raddeanin A (RA), an oleanane-type triterpenoid saponin derived from Anemone raddeana, reduces EMT and angiogenesis in GBM by down-regulating β-catenin expression.^135^ Periostin and anthocyanidins also inhibit EMT in GBM cells through the TGFβ/Smad2 signaling pathway.^136^^,^^137^ Paeoniflorin (PF), a polyphenolic compound derived from Radix Paeoniae Alba, suppresses EMT by inhibiting TGFβ signaling in GBM cells.^138^ Tetrandrine (TET) blocks MMP-2 and MMP-9 expression, thereby inhibiting the migration and invasion of GBM cells through suppression of MAPK signaling-induced EMT.^139^ Icaritin targets extracellular matrix metalloproteinase inducer (EMMPRIN) via the PTEN/AKT/HIF-1α pathway to suppress invasion and EMT in GBM cells.^140^ Chelerythrine inhibits the stemness of GBM by down-regulating the TGFB1-ERK1/2/Smad2/3-SNAIL/ZEB1 signaling axis.^141^ Galangin (GLN) targets Skp2-induced EMT and reduces CD44 expression, thereby suppressing migration, invasion, and angiogenesis in GBM cells.^142^^,^^143^ Kukoamine A inhibits proliferation and migration in GBM cells by down-regulating the expression of lipoxygenase-5 (5-LOX) and CCAT/ehancer binding protein beta (C/EBPβ), thereby suppressing EMT.^144^ Ginkgolic acid, which targets the chemokine monocyte chemoattractant protein-1 (MCP-1/CCL2), inhibits SNAIL and Slug expression via the JAK-STAT and PI3K-AKT signaling pathways, reducing the migration and invasion of GBM cells.^145^ Finally, Myrislignan, an NF-κB inhibitor, induces ferroptosis in GBM cells by regulating EMT through the Slug-SLC7A11 signaling pathway.^146^

### Antibody

The hepatocyte growth factor (HGF) antibody YYB-101 has been demonstrated to have inhibitory effects on the MET pathway in a glioma mouse model.^147^ When combined with temozolomide, YYB-101 significantly slows tumor progression and extends the survival of mice.^147^ In contrast, although the HGF antibody rilotumumab (AMG102) has shown some anti-tumor effects in preclinical trials, its combination with bevacizumab did not significantly inhibit tumor growth and resulted in high toxicity.^148^ Similarly, the MET monoclonal antibody onatuzumab exhibited anti-tumor effects in preclinical studies, but its efficacy in treating recurrent GBM patients has been limited.^149^

The PD-L1 pathway has been implicated in promoting GBM cell malignancy and aggressiveness, primarily through activation of the MEK/ERK pathway, which induces EMT.^150^ Clinical trials evaluating PD-1 and PD-L1 inhibitors in glioma are ongoing, with several in phases II and III. For instance, the phase III trial NCT02017717 investigated nivolumab in GBM, whereas the phase I/II trial NCT01952769 studied pidilizumab in diffuse intrinsic pontine glioma.^150^ In patients with recurrent GBM, the median OS was comparable between nivolumab and bevacizumab treatments.^151^

### Small molecule inhibitors

Crizotinib is a small-molecule inhibitor approved by the US Food and Drug Administration for the treatment of anaplastic lymphoma kinase (ALK) mutations.^152^ In addition to ALK, it also inhibits the kinase activity of MET and ROS1.^153^ A clinical trial investigating crizotinib as an adjuvant therapy for adult GBM is currently underway (NCT02270034).^154^ Cabozantinib, which simultaneously targets MET and VEGFR2, has demonstrated limited inhibitory effects on tumors in clinical trials for GBM patients who are not receiving anti-angiogenic therapy (NCT00704288).^155^ The MET pathway and its downstream STAT3 signaling are activated in human astrocytes by MET exon 14 skipping (METex14) and PTPRZ1-MET fusion.^156^ Bozitinib (PLB-1001), a MET kinase inhibitor with good blood‒brain barrier permeability, is considered a promising therapeutic option for treating intracranial tumors.^156^ Metformin has been shown to inhibit TGF-β1-induced EMT and stemness in GBM cells through the AKT/mTOR/ZEB1 pathway.^157^ Foretinib, a c-MET inhibitor, induces cell cycle arrest, apoptosis, and reduces invasion in GBM cells through its effect on c-MET.^158^ The cyclopeptide moroidin significantly inhibits migration and vasculogenic mimicry (VM) formation and reduces the expression of α-smooth muscle actin and MMP-9 in human GBM cells by blocking the ERK/β-catenin-mediated EMT.^159^ Ticagrelor inhibits GTSE1-induced EMT in GBM cells by suppressing the PI3K/AKT/NF-κB signaling pathway.^160^ SC66, an AKT inhibitor, downregulates the AKT/β-catenin signaling pathway, inhibiting EMT-driven cell migration and invasion in GBM cells.^161^ CYY292, a novel FGFR1 inhibitor, suppresses GBM progression, invasion, and metastasis by targeting the Akt/GSK3β/SNAIL signaling axis.^162^ YM155, a survivin inhibitor, decreases radiation-induced invasion and reverses EMT by targeting STAT3 in GBM cells.^163^ A new STAT3 inhibitor, HJC0152 (also known as Bt354), exhibits antitumor activity in GBM by suppressing TWIST1, vimentin, N-cadherin, and MMP2/9 expression.^164^^,^^165^

The correlation between drug sensitivity and the mRNA expression of EMT-related genes is summarized in Figure S4 and is based on data from the Genomics of Drug Sensitivity in Cancer (GDSC).

### Chemical agent

3-Benzyl-5-((2-nitrophenoxy)methyl)dihydrofuran-2(3H)-one (3BDO), an autophagy inhibitor, suppresses proliferation, EMT, and stemness in GBM cells by targeting survivin 166. Enhydrin inhibits EMT by modulating the Jun/Smad7/TGF-β1 signaling pathway in GBM cells.^167^ d-Penicillamine (DPA) reduces proliferation and inhibits EMT through the TGF-β/Smad signaling pathway in GBM cells.^168^ Ethyl pyruvate suppresses migration and invasion by modulating NF-κB- and ERK-mediated EMT in GBM cells.^169^ Conversely, 17β-estradiol (E2)-induced EMT enhances the migration of GBM cells through estrogen receptor-α (ER-α) signaling.^170^

### Limitations

Despite the promising potential of EMT-targeted therapies in GBM, several critical clinical limitations hinder their efficacy and broader clinical application. A major challenge is restricted penetration of the blood‒brain barrier (BBB).^171^ Many small molecules, antibodies, and chemical agents struggle to traverse the BBB efficiently, leading to reduced bioavailability and diminished therapeutic efficacy in brain tumors. Even for inhibitors with BBB permeability, their intratumoral concentrations may remain suboptimal for achieving therapeutic success. Moreover, GBM cells exhibit remarkable plasticity, enabling them to evade EMT-targeted therapies through compensatory signaling mechanisms.^172^ Resistance pathways, including alternative activation of Wnt, PI3K/AKT, and NF-κB signaling, can significantly undermine the long-term effectiveness of EMT inhibitors. While targeting EMT holds promise for limiting GBM progression, addressing these clinical challenges remains a formidable task. Future research should prioritize the development of more selective inhibitors, the optimization of combination therapies, and the identification of reliable biomarkers for improved patient stratification. Additionally, advanced drug delivery strategies, such as nanoparticle-based approaches, may help overcome BBB penetration barriers, ultimately enhancing the efficacy of EMT-targeted treatments in GBM.

## Conclusion

EMT plays a critical role in tumor initiation, progression, and response to treatment. In GBM, the activation of EMT is often linked to various signaling pathways, including the TGF-β, Wnt, Notch, and EGFR pathways. Despite its significant role in GBM progression, targeting EMT as a therapeutic strategy presents several challenges. One major difficulty is that EMT contributes to the maintenance and expansion of cancer stem-like cells within GBM tumors, which are relatively resistant to conventional therapies. This cellular plasticity makes it difficult to achieve sustained therapeutic effects by targeting EMT alone. Therefore, future research should focus on further elucidating the molecular drivers of EMT in GBM and developing combination therapies that can effectively disrupt this process, overcome resistance, and enhance the immune response.

## Funding

This review was supported by the 10.13039/501100001809National Natural Science Foundation of China (No. 81972784).

## Conflict of interests

The authors declared no competing interests.
