# Supplementary material for: The significance of epithelial–mesenchymal transition (EMT) in the initiation, plasticity, and treatment of glioblastoma
Source: Genes Dis. 2025 Jun 6;13(1):101711. doi: 10.1016/j.gendis.2025.101711 (PMC12547761; doi:10.1016/j.gendis.2025.101711)
Supplement: Multimedia component 1 [file mmc1.docx]

A


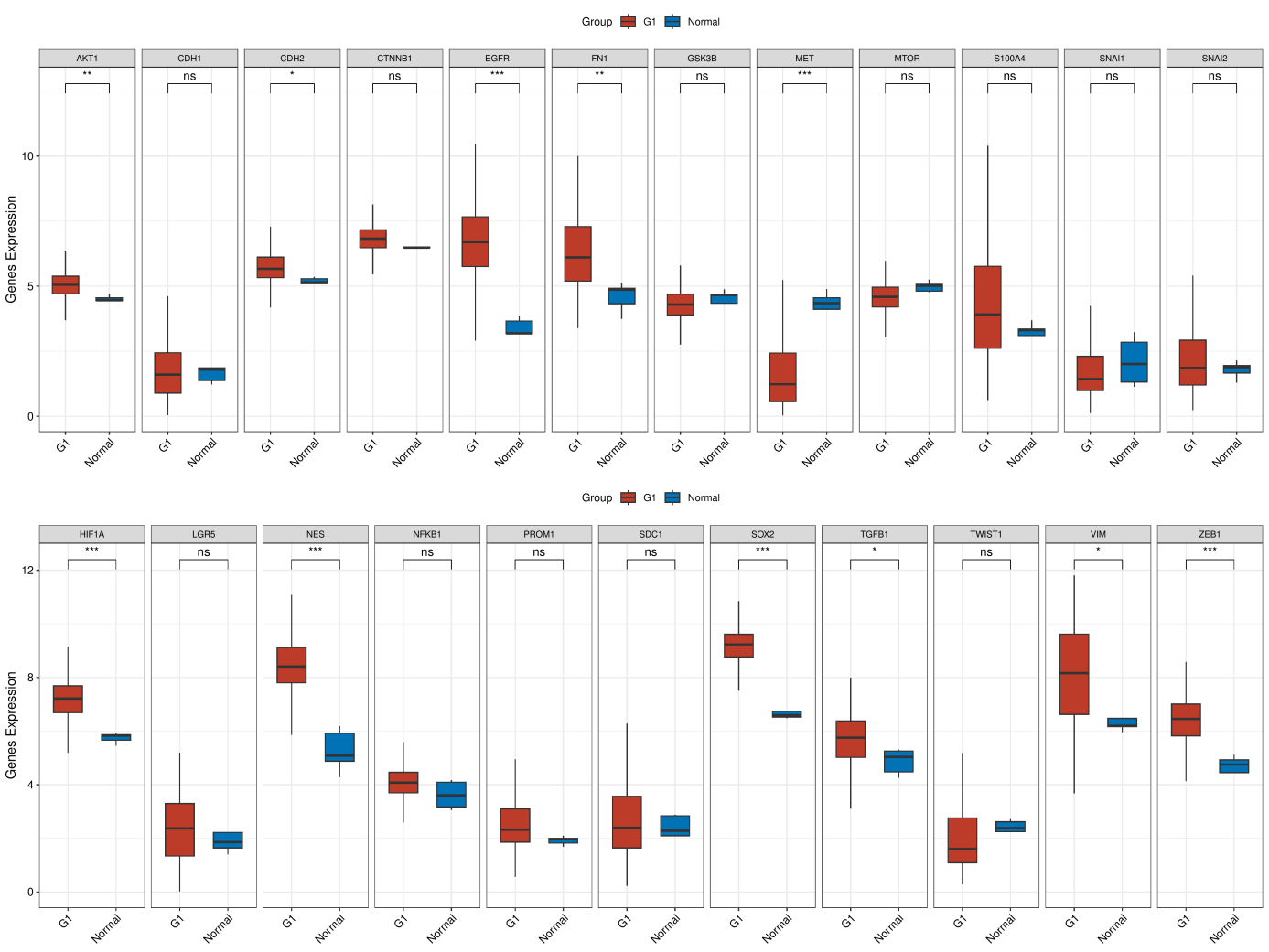


B


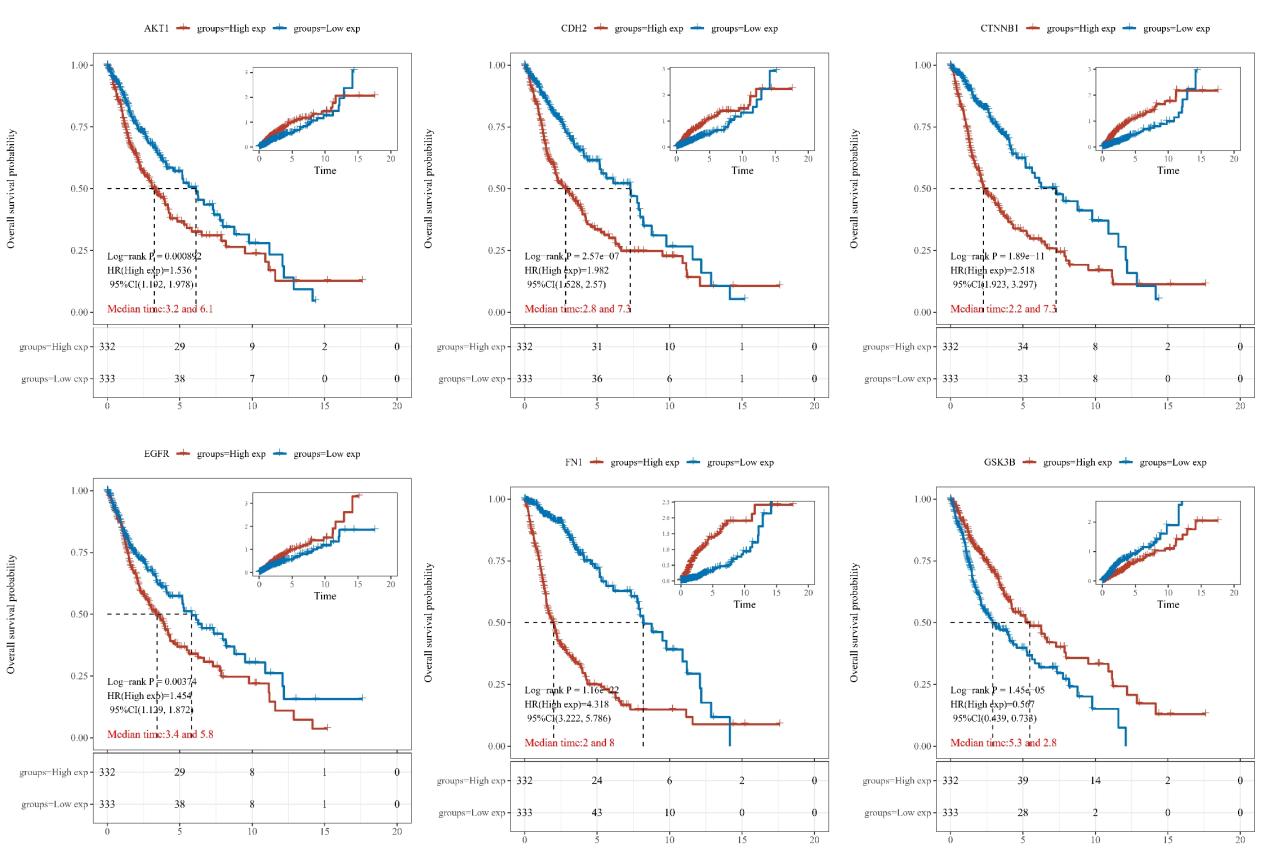


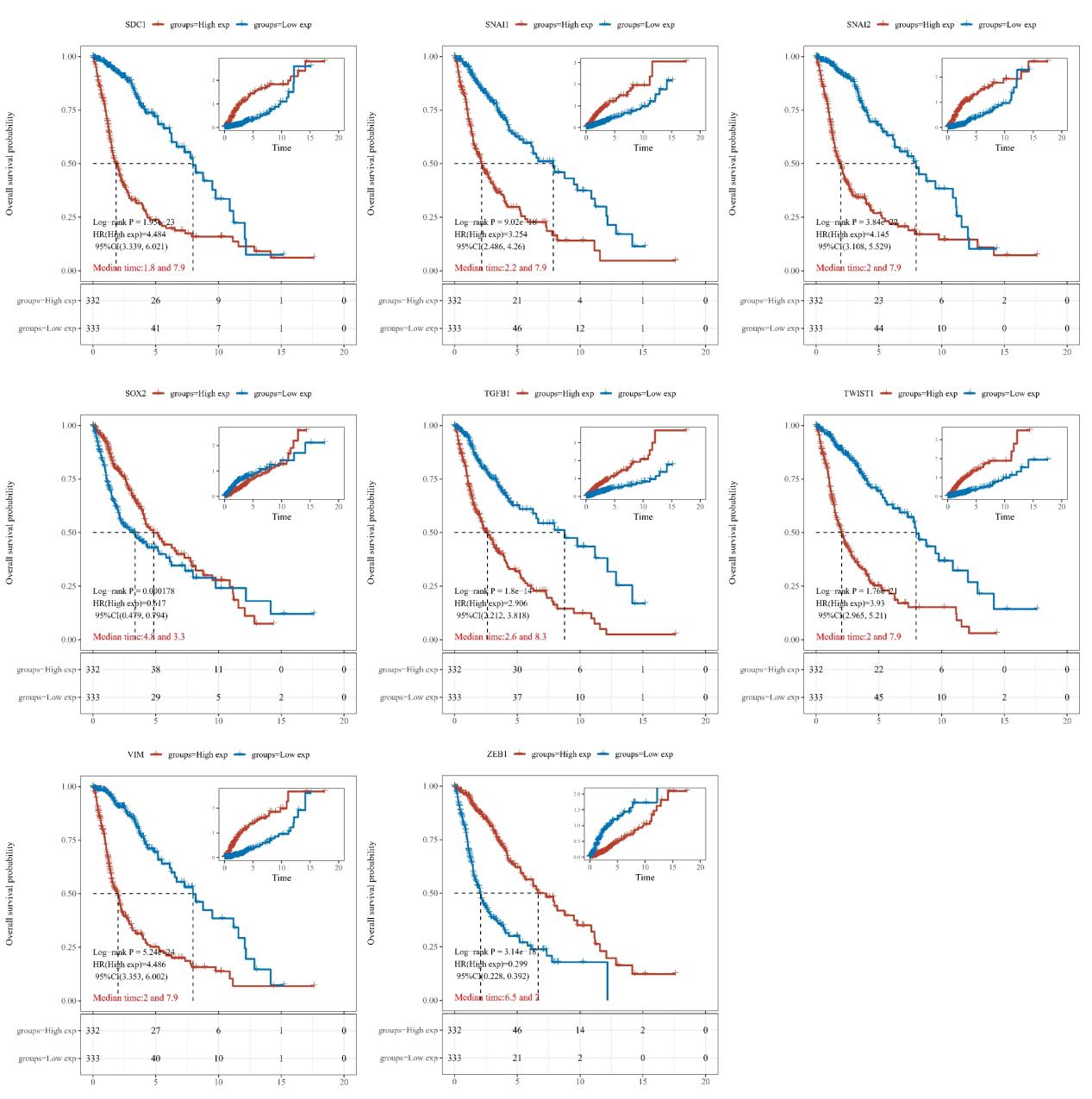

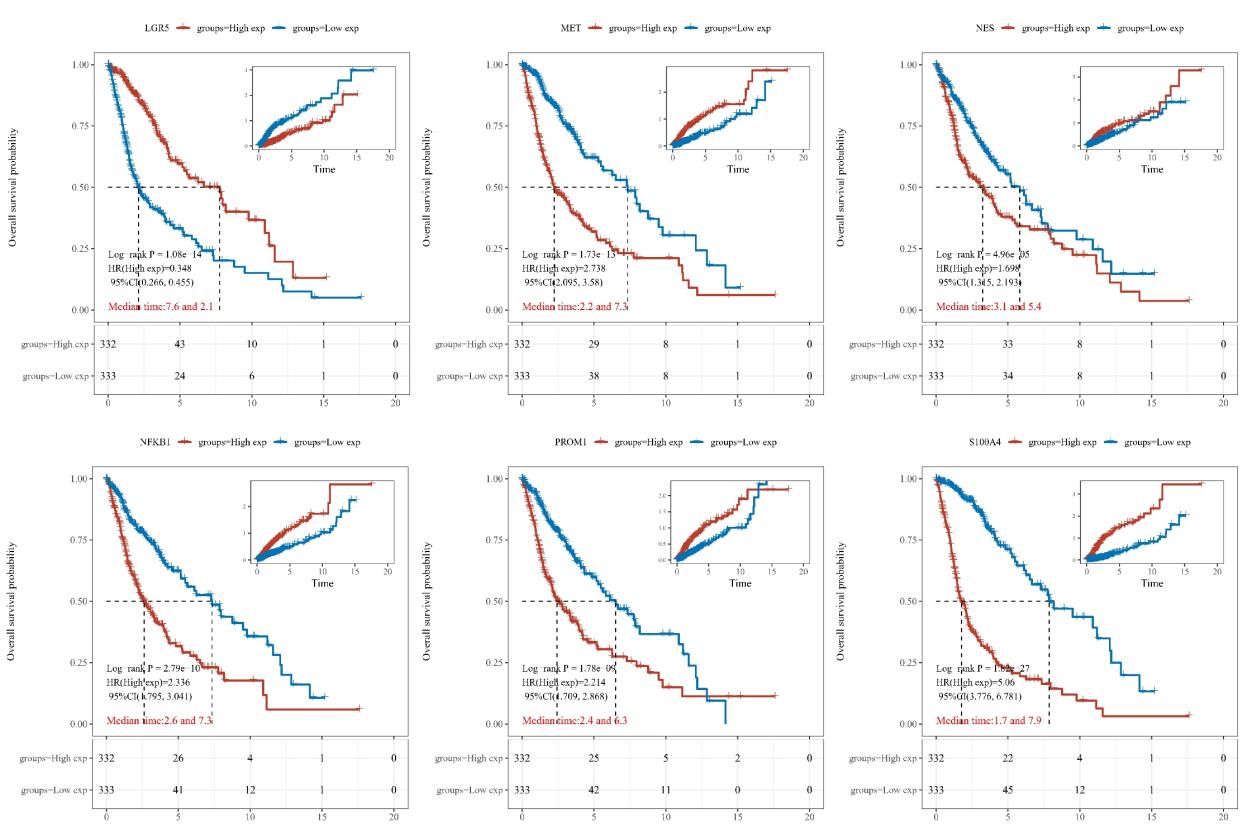


Supplementary Figure 1. (A) Expression levels of genes related to EMT and glioblastoma stem cell (GSC) markers in normal and cancerous samples from glioblastoma (GBM) patients.

(B) Kaplan-Meier survival analysis showing the relationship between these genes and overall survival (OS) time in GBM patients.
