# Supplementary material for: The significance of epithelial–mesenchymal transition (EMT) in the initiation, plasticity, and treatment of glioblastoma
Source: Genes Dis. 2025 Jun 6;13(1):101711. doi: 10.1016/j.gendis.2025.101711 (PMC12547761; doi:10.1016/j.gendis.2025.101711)
Supplement: Multimedia component 2 [file mmc2.docx]

A


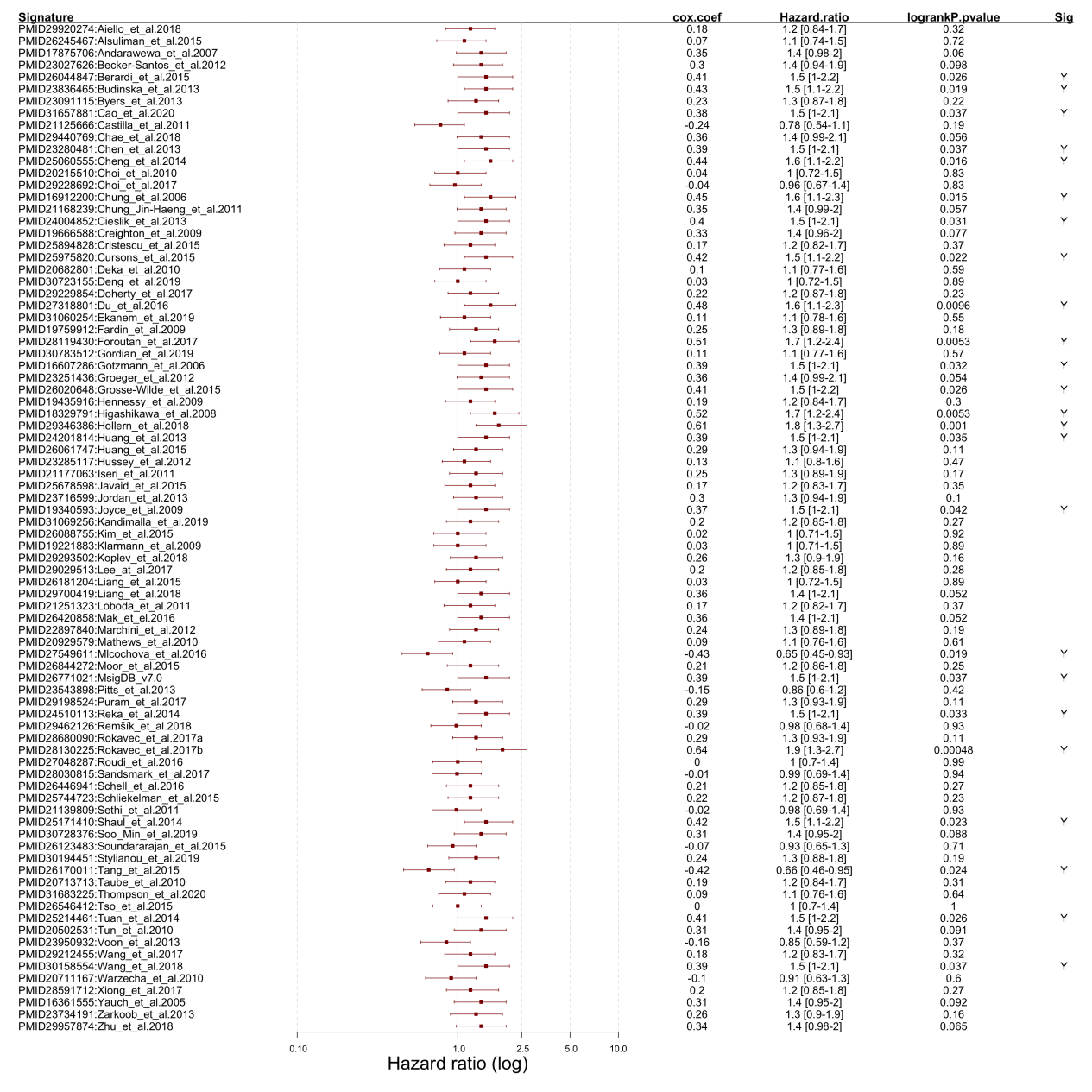


B


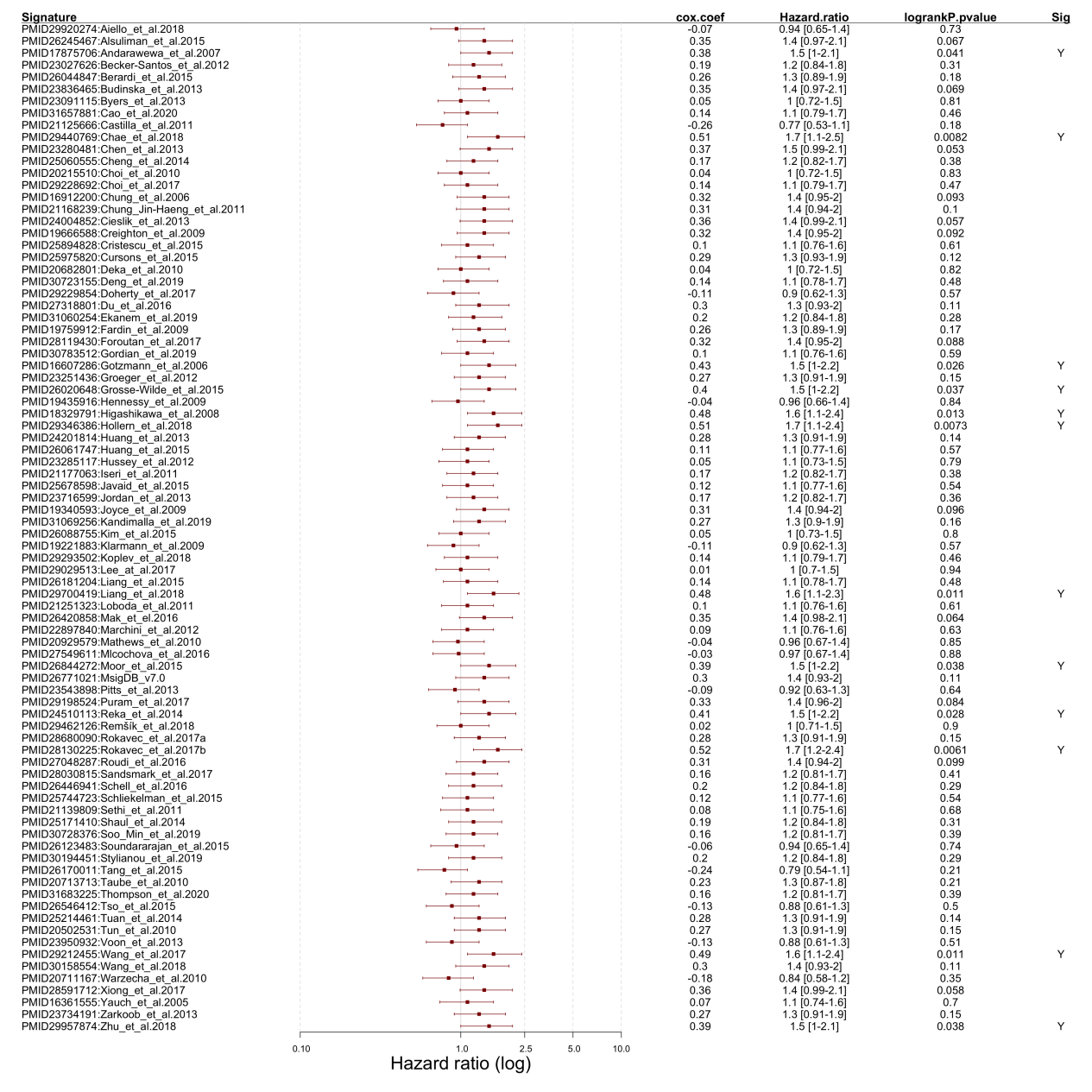


Supplementary Figure 2. Comparative Progression free (A) and overall survival (B) analysis of EMT-signatures based on cumulative EMT-signature gene set at the patient level in a glioblastome cohort shown as a survival forest plot.
