# Supplementary material for: The significance of epithelial–mesenchymal transition (EMT) in the initiation, plasticity, and treatment of glioblastoma
Source: Genes Dis. 2025 Jun 6;13(1):101711. doi: 10.1016/j.gendis.2025.101711 (PMC12547761; doi:10.1016/j.gendis.2025.101711)
Supplement: Multimedia component 3 [file mmc3.docx]

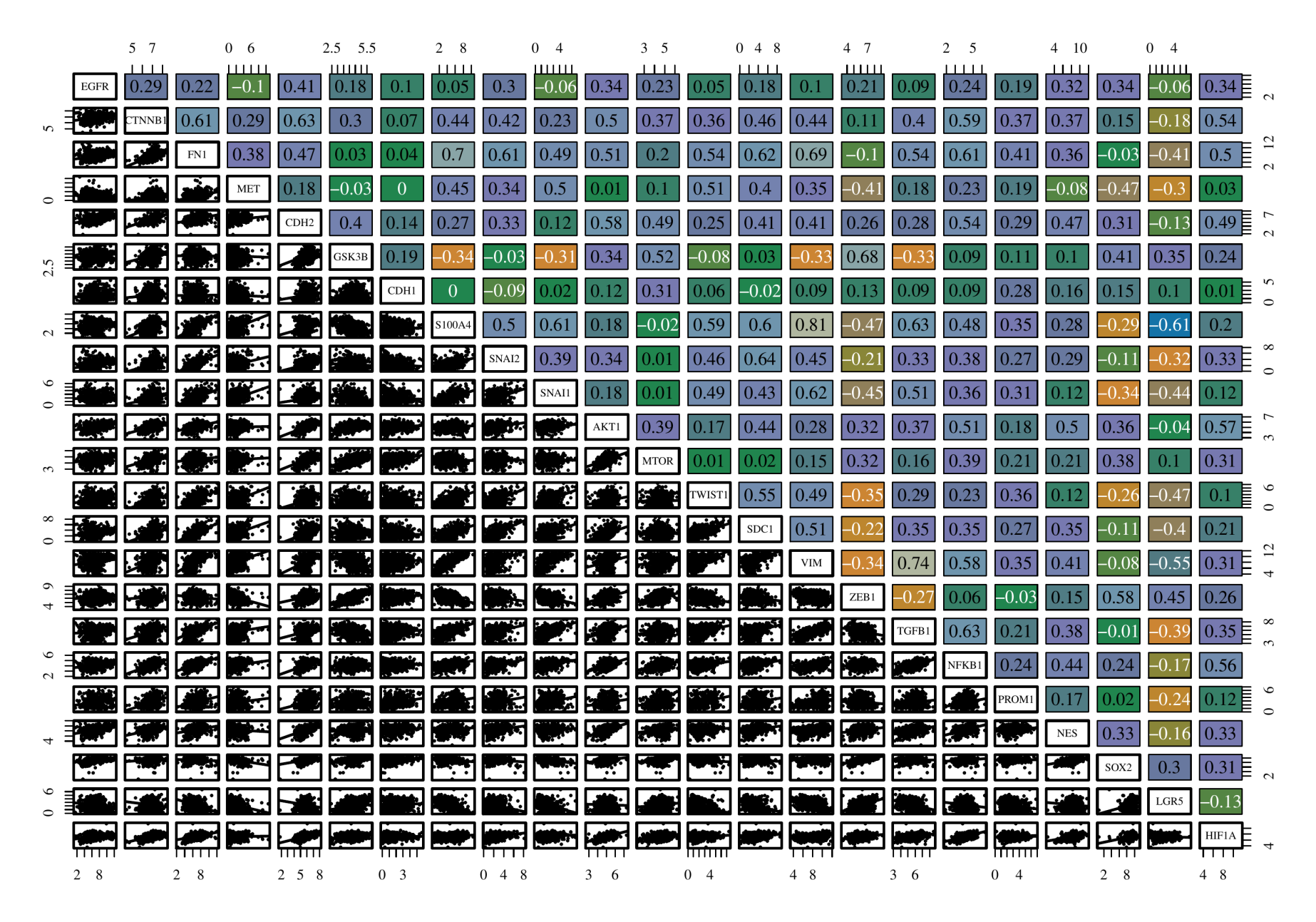


Supplementary Figure 3. Multi-gene correlation map of genes related to EMT and glioblastoma stem cell (GSC) markers was generated using the R package “ggstatsplot” and visualized with the “pheatmap” package.
