# Supplementary material for: The significance of epithelial–mesenchymal transition (EMT) in the initiation, plasticity, and treatment of glioblastoma
Source: Genes Dis. 2025 Jun 6;13(1):101711. doi: 10.1016/j.gendis.2025.101711 (PMC12547761; doi:10.1016/j.gendis.2025.101711)
Supplement: Multimedia component 4 [file mmc4.docx]

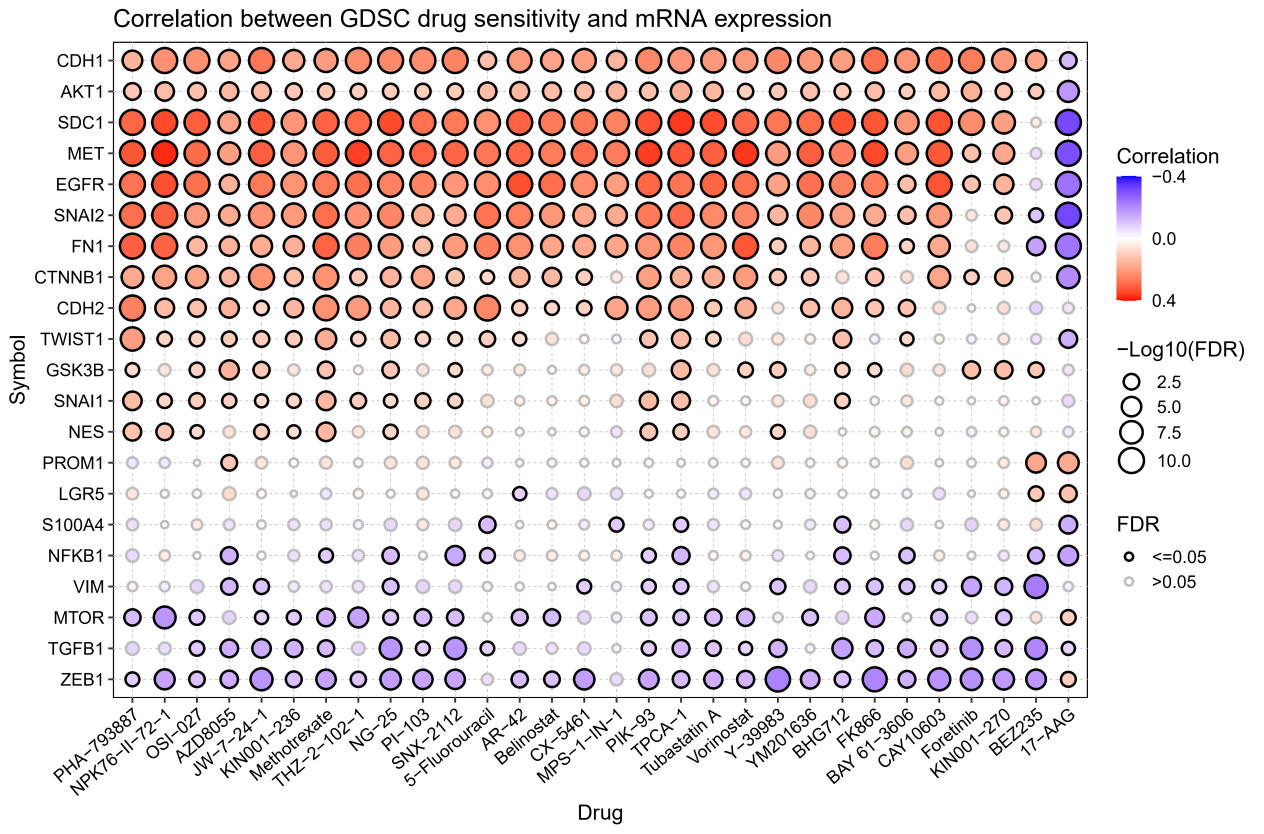


Supplementary Figure 4. Correlation between drug sensitivity data from the Genomics of Drug Sensitivity in Cancer (GDSC) and the mRNA expression of genes associated with EMT. The data were obtained using the GSCALite platform.
